# The research priorities of patients attending UK cancer treatment centres: findings from a modified nominal group study

**DOI:** 10.1038/sj.bjc.6603662

**Published:** 2007-03-06

**Authors:** J Corner, D Wright, J Hopkinson, Y Gunaratnam, J W McDonald, C Foster

**Affiliations:** 1 School of Nursing and Midwifery, University of Southampton, Southampton SO17 1BJ, UK; 2 Macmillan Research Unit, School of Nursing and Midwifery, University of Southampton, Southampton SO17 1BJ, UK; 3 Division of Social Statistics, School of Social Sciences and Southampton Statistical Sciences Research Institute, University of Southampton, Southampton SO17 1BJ, UK

**Keywords:** consultation, service users, cancer research agenda, participatory, priorities

## Abstract

Members of the public are increasingly consulted over health care and research priorities. Patient involvement in determining cancer research priorities, however, has remained underdeveloped. This paper presents the findings of the first consultation to be conducted with UK cancer patients concerning research priorities. The study adopted a participatory approach using a collaborative model that sought joint ownership of the study with people affected by cancer. An exploratory, qualitative approach was used. Consultation groups were the main method, combining focus group and nominal group techniques. Seventeen groups were held with a total of 105 patients broadly representative of the UK cancer population. Fifteen areas for research were identified. Top priority areas included the impact cancer has on life, how to live with cancer and related support issues; risk factors and causes of cancer; early detection and prevention. Although biological and treatment related aspects of science were identified as important, patients rated the management of practical, social and emotional issues as a higher priority. There is a mismatch between the research priorities identified by participants and the current UK research portfolio. Current research activity should be broadened to reflect the priorities of people affected by the disease.

Patient engagement in the development and monitoring of health services and in the conduct of health research has increased in importance in recent decades. The UK government's commitment to patient involvement is evident with the publication of documents such as Choosing Health and Our health, our care, our say (Department of Health, 2004, 2006). Similarly, the involvement of service users is seen to be an important part of good research practice (Department of Health, 2005) and the advantages of patient engagement are well documented (Gray *et al*, 2000; Tallon *et al*, 2000; Hanley *et al*, 2003). Recent guidelines have recommended involving service users at every stage of the research process from identifying research priorities through data collection and analysis to the dissemination of findings (Hanley *et al*, 2003). Involving patients in setting the research agenda is particularly important as their views may differ from current research practice and are more likely to reflect the interests of the general public and thus health care, public health and social care services (Tallon *et al*, 2000; Hanley *et al*, 2003). Furthermore, the legitimacy and sustainability of investment decisions made by research funding bodies will increasingly depend on how well they reflect the underlying values of the public (Maxwell *et al*, 2003).

Patient engagement in determining research priorities remains a developing field of study, particularly in cancer research (Lomas *et al*, 2003; Corner and Wright, 2004; Oliver *et al*, 2004). To date, there has been no comprehensive attempt to elicit patients' views to inform the strategic direction for cancer research on a UK wide basis (Corner and Wright, 2004). Strategies for research funding decision-making are multifaceted and involve considerations such as the critical appraisal of research questions and the relative importance of the studies. It has been suggested that economic constraints result in a preference for cost–benefit analyses above patient-derived prioritisation methods (Stewart, 1995; Liberati, 1997). However, there is growing recognition that research has to respond to the needs of service users as well as the professionals' agenda (Tierney, 1998).

Priorities for cancer research in the UK have traditionally been determined by individual funding bodies, usually involving the scientific community. Questions have been raised in recent years over the extent to which people affected by cancer should be involved in setting the research agenda. A study conducted by the US National Cancer Institute, for example, found that patients were critical of how research priorities are determined despite being supportive of cancer research (Jenks, 1997). Similarly in the UK, involving people affected by cancer in setting research priorities has been seen to be a means of improving public confidence in cancer research (Glass, 2002).

In light of the limited understanding of the research agendas of people affected by cancer, Macmillan Cancer Support initiated the first study to involve cancer patients across the UK in identifying priorities for research investment. The study was supported by the UK National Cancer Research Institute. This paper presents the key findings from this work.

## MATERIALS AND METHODS

Given the interest in patient derived priorities for research, it was considered appropriate to adopt a participatory approach as the theoretical frame of reference for the study (Parkes and Panelli, 2001; Reason and Bradbury, 2001; Wright *et al*, 2006). Participatory research sees research as a democratic process where participants are active citizens rather than passive ‘subjects’ (Reason and Bradbury, 2001). Consequently, all elements of the study were developed and designed in consultation with a reference group comprising patient and carer representatives and a stakeholder group representing patient interest groups and major research funding bodies. Patient and carer representatives were approached through the user partnership groups of the cancer networks located across the UK. In addition, community representatives from under-researched sections of society were also involved through participating sites and national organisations. Fifteen patients and carers from the reference group became co-researchers and received training and support to collect data and conduct analysis with the Macmillan Research Unit (Wright *et al*, 2006). The co-researchers ‘co-owned’ the study with the Unit, and as such had a direct influence on all aspects of the study, including data collection, analysis and dissemination of study findings. Inevitably the level of research knowledge and experience differed between co-researchers and professional researchers, and this was managed through the ongoing negotiation of the respective roles and responsibilities; co-researchers undertook greater responsibility as they gained in confidence and expertise. The participatory approach was successful as a vehicle to engaging cancer patients in discussions about research priorities, and participants valued the data collection being co-led by someone who had experienced cancer themselves. A more detailed description of their involvement in the study has been published elsewhere (Wright *et al*, 2006).

Consultation groups were the main method of data collection, which combined a focus group approach with an adapted Nominal Group Technique. Focus groups were appropriate as they allowed patients themselves to set the boundaries of what they perceived to be legitimate avenues for research. This is necessary in studies where limited research evidence necessitates an exploratory approach (Krueger, 1994). However, unlike typical focus groups, the discussion was structured by using an amended Nominal Group Technique (NGT). The amended NGT was adopted in order to achieve consensus over research priorities. Nominal Group Techniques were developed to aid community decision-making and are particularly helpful in generating ideas and priorities in situations where participants are likely to have diverse views on a subject where little is known (Murphy *et al*, 1998). They also prevent dominant participants from controlling the group discussion and allow participants to raise views and opinions in a manner protected from the direct rejection of other participants (Wellings *et al*, 2000).

**Figure 1:**
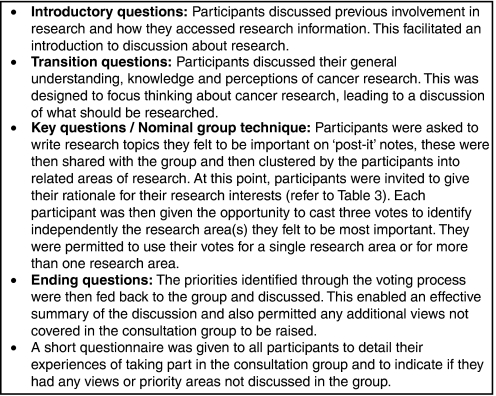
Process of identifying research priorities in consultation groups.

Nominal Group Techniques have been successfully applied to various areas of health research (Campbell and Cantrill, 2001; Telford *et al*, 2004) and have been used specifically to identify research priorities (Duncan *et al*, 2003). Nominal groups typically involve participants recording ideas independently and in private, then sharing, listing and discussing the ideas, and finally judging or voting on the ideas independently. It is usual for participants to be asked to identify issues before attending the NGT session. However, as Aspinal *et al* (2006) discuss, in studies where sensitive issues may be raised, it is preferable to modify the NGT to limit prior preparation. Thus, all ideas were generated within the group to reflect the particular needs of the participants. Figure 1 details the format of group discussions.

Consultation groups lasted approximately 1.5–2 h and were held at a mutually convenient local venue, such as the cancer centre. A stakeholder panel advised on the nature of the consultation groups, as recommended by Lomas *et al* (2003). A patient/carer co-researcher and a member of the research team jointly moderated the consultation groups. Another patient/carer co-researcher acted as an observer in most consultation groups. Group discussions were tape recorded and subsequently transcribed verbatim. Training for the co-researchers was extensive and involved issues such as avoiding leading questions, encouraging discussion from all participants and being reflective of the potential impact of the researcher on the discussion, thus helping to maintain the quality of the data (Wright *et al*, 2006).

Ethical approval for the study was gained through the South East Multi-Centre Research Ethics Committee. Local Research and Development Governance approval was secured according to local requirements in each study setting. All participants gave written consent to participate.

### Participants

The study sought to elicit the views of ‘unorganised’ (as opposed to self-selecting and ‘expert’) cancer patients (Maxwell *et al*, 2003). Hence, participants (⩾18 years) were recruited through outpatient clinics in seven cancer centres across the UK on a sequential basis using a population sample stratified by gender and stage of treatment. There was a maximum of four breast cancer patients per group so that a broad range of demographic characteristics and cancer types were represented. Patients were excluded from the study if they were deemed by the research nurse or other members of the clinical team to be too unwell, have complicating health factors or liable to be distressed by study participation. All other potential participants were invited to participate regardless of prior involvement in research.

To ensure that people from diverse communities and backgrounds were included, consultation groups were also run with purposively selected participants from frequently under-researched communities. These included two consultation groups with participants from a South Asian cancer support group conducted in English, Hindi and Gujarati, a consultation group with people aged over 75 years and two consultation groups with people with advanced cancer recruited from day care services in two hospices.

### Analysis

As the purpose of the study was to reach consensus over priorities for cancer research through the application of a nominal group technique, this was the focus for the analysis. The consultation groups generated two forms of data: a ranked list of research topics and questions arranged into themes within each consultation group by participants, and transcripts of recorded discussion. The transcripts provided contextual data regarding the meaning of research topics as well as participants' rationale for identifying them as important.

To develop a composite list of research themes across all 17 consultation groups, a process for grouping similar clusters of research topics was undertaken using a form of thematic analysis (Strauss, 1987). DW analysed all 17 research priority lists generating a name for each cluster of research topics. Nested themes were identified under higher order themes. The reliability of the analysis was assessed through a process of independent assessment of all priority lists involving two members of the research team and five patient/carer co-researchers (four involved in data collection and one not). Transcripts of consultation groups were consulted where research ideas were unclear. With few exceptions, the independent analysts agreed on the names generated.

The ranked lists of research themes for the 17 consultation groups were then combined taking into account the ranked scores of each theme within each group. The ranked scores within each consultation group have a possible range from 1 (for the research theme receiving the least votes) to 6 (for the research theme receiving the most votes. Six was the maximum number of research theme clusters identified during the vote casting exercise in any consultation group). Therefore, the highest score possible from this exercise across all 17 groups is 102 (6 × 17=102) for any given theme. A higher score indicates a larger number of votes.

The consultation group transcripts were also subjected to thematic analysis. This was appropriate given the need to contextualise the identified research priorities. Data analysis occurred iteratively and a process of progressive focussing occurred whereby topics identified through an initial reading of the interview transcripts were clustered into a set of emerging themes (Hammersley and Atkinson, 1989). After this, the range of responses relating to individual themes was identified and then organised into subthemes. DW analysed all transcripts and was verified by the co-researchers and through independent analysis of six transcripts by two researchers who met initially to devise a coding framework and then continued to analyse a subset of the transcripts. Again, with few exceptions, there was close agreement between the analyses. Data were managed and retrieved using NUD^*^IST 6 software.

## RESULTS

### Participants

A total of 17 consultation groups were conducted with 105 participants with a median of 6 (range 3–11) participants in each. An audit of recruitment was undertaken in six participating centres. Across these sites, 379 patients were approached of which 80 (21.1%) participated in the consultation groups. Men were more likely to decline, almost one-third of patients were male, despite being oversampled during recruitment (*n*=33, 31.4%). A common reason for refusal was not wishing to share views in a group.

Participants had a range of cancer types and sites and 11.4% of participants were from Black Caribbean and South Asian Groups (*n*=12) (Table 1). Forty-six participants (43.8%) indicated that they had been involved in research before taking part in the study, most of who had been involved in clinical drug trials.

### Research priorities

Fifteen research themes were generated in the consultation groups (Table 2). Table 3 illustrates each of these themes with reference to excerpts from consultation group transcripts. In most consultation groups, consensus over research priorities was easily attained. There were rare occasions, however, where consensus was not reached. These were documented in the analysis.

#### Impact on life, how to live with cancer and related support issues

The highest priority for future research identified by consultation group participants was research into the ‘impact on life, how to live with cancer and related support issues’. This broad theme included psychological consequences of cancer (impact on the patient or others, the influence of mental attitude on recovery); support groups (evidence of effectiveness) and after-care (the need for improved after care as well as outcomes of care); the impact cancer has on social functioning (such as driving, travelling, shopping); employment and financial issues (difficulty for patients gaining employment, re-entering the workplace or continuing work following treatment, the financial cost of cancer in terms of treatment, insurance and benefits).

There was little discussion of symptoms, although pain management was raised in several consultation groups and in one palliative care group in particular. Greater priority was given to how patients could manage cancer themselves, particularly in the area of diet and general lifestyle.

Group discussions revealed the rationale for prioritising research on the impact of cancer on life so highly. It was felt important to document the challenges experienced by cancer patients and their families, and to identify means of improving quality of life and the quality of care:

‘I would love for somebody to come and… find out how we are all coping and the reasons why we are coping. What are the reasons why we are not coping? Could it be that we are having bad experiences in hospital?. Could it be that we are having difficulties at home because our partners can't cope and are taking it out on us in some way?. And so, just that small study would be of enormous interest, I think, to the people who plan and deliver our care.’ (Linda, Breast Cancer Patient, Mixed Consultation Group)

Participants believed the emphasis in research had centred on developing cancer treatments whereas the experience of managing the impact of cancer on individuals was felt to have been relatively neglected. In contrast, though most participants had undergone cancer treatment, research into cancer treatment was ranked seventh alongside research into the management of side effects of treatment and different cancer types. Participants often felt that, while the drive of medical research was to discover new treatments for cancer, research into the cost of cancer and cancer treatment to the individual received less attention. Hence, participants called for a balance in research effort so that the personal consequences of cancer are also addressed.

#### Risk factors and causes

Risk factors and causes was the second highest priority. Four main areas of concern were raised: the environment, genetics, diet and stress (Table 3). Environmental issues related to general concerns, such as air pollution, electricity pylons and nuclear power stations and the everyday use of potentially harmful devices, such as mobile phones, TVs, computers, microwave ovens and aerosols. Diet was raised as a possible causal factor in cancer and also whether periods of high anxiety and stress could be a contributing factor. A common question raised was whether family members needed to be tested after a diagnosis of cancer and to know if their cancer diagnosis was related to previous cancers in the family. Research into genetics and family history was related to early detection and screening.

Risk factors and causes was a priority for participants as they had a personal interest in what caused their cancer and believed that identifying causes was an essential part of preventing cancer. Participants often justified this priority by reflecting on potential causes for their own cancer:

‘There must be ongoing research into these electricity pylons… I know a particular pylon where I used to live… A friend of mine who moved from there, he's got prostate cancer, a lady who bought his house from him near that pylon died from cancer. Just up the road from where I used to live, the next house up, the husband and the wife in their early fifties both had Hodgkin's or non Hodgkin's. Somebody else in that road had cancer, I live sixty yards down the road, I've had cancer. What's in the ground, is there a stream with something running through?’ (Jim, Bowel Cancer Patient, Mixed Group)

#### Early detection and prevention

Early detection and prevention was the third highest priority theme. Cancer prevention was seen in three ways: an avoidance of risk factors, the detection of cancer at a precancerous stage and the prevention of cancer from advancing to a more aggressive state after diagnosis (Table 3). Participants suggested that research should identify and develop measures and techniques to identify particular cancers easily and effectively. A significant area of concern was the role of primary care and the GP in detecting cancer early. Research into diet as a means of cancer prevention was identified. In particular, there was a view that certain food types may prevent cancer and participants wanted to see research evidence to support this.

Many participants felt that preventative research should take precedence over other types of research, as ‘prevention is better than cure’. Cancer prevention was prioritised as it was felt that research into this area would prevent the impact of the disease on the patient and the resource implications of treatment:

A cure could be very expensive, lots of medical resources, but prevention, if you can nip a thing before it even starts, nip it in the bud, it's much better than having to go into hospital and maybe having major surgery and all the follow-up treatment that you need. (Stella, Breast Cancer Patient, Mixed Consultation Group)

## DISCUSSION

Our study illustrates that, contrary to common perceptions (Hanley *et al*, 2003), cancer patients are able to engage with a broad range of issues relating to science, medicine, health and social care, the purpose and value of cancer research and can identify and agree on priorities. The highest priority area did not differ markedly across groups, including those that specifically targeted older patients, patients from South Asian communities or patients with advanced cancer.

Our findings reflect those of research prioritisation studies conducted in areas other than cancer in that the priorities of patients differed from current research activity. Two of the three highest priorities for patients identified in this study comprise around 4% (supportive and palliative care) and 12% (early detection and prevention) of UK cancer research funding, with cancer biology comprising 43% (National Cancer Research Institute, 2004). Patients' concerns primarily lie with the need for assistance with what it means to live day to day with cancer, which they feel is lacking, and not, as one might expect, with finding the most effective treatments for cancer. The reason for the emphasis placed on how cancer impacts on daily life may be because patients are well aware of the significant activity underway in research into new cancer treatments, but feel that their other needs are not being addressed.

Decisions around investment in research by funding bodies has, to date, largely been determined through the generation of research topics from within the biomedical community and this accounts for the relative imbalance in research monies allocated to addressing the priorities of patients. There are also few incentives for pursuing research into the impact of cancer on life as discoveries or developments in this area are not high profile in academic or scientific communities and do not lead to inventions or technologies that can be exploited commercially. This may also account for why a relatively small proportion of funding is allocated to the public health imperatives of causation, prevention and screening. The involvement of patients in setting priorities for research funding might involve a significantly increased investment in these areas.

Although a large body of research has been undertaken into the psychological consequences of cancer and into quality of life during cancer treatment, this clearly has not been made available to people with cancer themselves as this study reveals they are not aware of its existence.

The second priority area, ‘risk factors and causes’, has some parallels with the greatest area of research activity in the UK (cancer biology). However, patients refer to concerns relating to environmental and hereditary factors rather than research into the biology of carcinogenesis *per se* although this may be due to the highly specialised nature of cancer science, knowledge of which is not available to most lay people. A need to develop greater understanding of cancer science among the public and patients is indicated.

The findings of this study support those of the US National Cancer Institute; some patients are critical about how research priorities are made (Jenks, 1997). There are parallels here with AIDS activism where the boundaries of science and motivations of the medical and scientific communities have been challenged by patients (Epstein, 1996, 1997). As in the case of AIDS, this study raises the question as to who should have a seat at the table when determining decisions about investment in cancer research and demands that cancer research is made more democratic and more accessible to the communities it seeks to serve (Martin, 1980; Porter, 1997). It has been suggested that engaging with communities more effectively can rectify the mistrust of research and thus improve the conduct and outcomes of research (Newman, 2006).

### Limitations

The study has limitations. Given the paucity of literature on the research priorities of people affected by cancer, it was necessary to undertake an exploratory study. This was successful in allowing participants to raise their own issues but limited the population sample of the study. Further research is therefore required to assess the generalisability of findings across a larger population. In particular, 20% of patients, fewer men than women and few patients with aggressive tumours, such as lung or pancreatic cancer, participated in the consultation. Participants from minority ethnic backgrounds were underrepresented in the recruitment from cancer centres. Although South Asian participants were recruited through patient support groups, these participants were less typical than patients recruited through the centres. Participants were patients attending cancer treatment settings as well as palliative care services, and while there was substantial consensus over research priorities among the range of patients who participated it may be that individual views change over time. This study did not set out to ascertain the views of people who do not have cancer or people who have been bereaved as a result of cancer, nor does it reflect the views of patients in other contexts such as resource poor countries. It may be important to know how the views of such individuals differ from the perspectives of these patients. Although consultation groups led by patients and carers were highly valued by participants, it is possible that this influenced the discussion in consultation groups and in turn the topics generated as priorities.

## CONCLUSION

This study indicates the importance of involving patients and the public in the identification of strategic priorities for research. As the beneficiaries of discoveries and developments in cancer science, it cannot be assumed that their views will automatically accord with those of the scientific community. It would appear that patients with cancer, regardless of their personal situation have clear views as to the most important priorities for research investment. Their highest ranked concerns are not currently being addressed. In addition, dissemination of research findings to patients is currently inadequate.

A clear direction for the future agenda for cancer research is indicated whereby a better balance is achieved between research into the causes of the disease and treatments and research that helps people to live with illness and its consequences. Furthermore, it is evident there is a need to consider the views of patients alongside traditional strategies for research prioritisation when making funding decisions in the future. Research funding bodies and the scientific community must consider how they will respond to the priorities of patients.

## Figures and Tables

**Table 1:** Participant data

**Participant data**	***N*=105 (%)**
*Gender*	
Male	33 (31.4)
Female	72 (68.6)

*Age*	
30–39	4 (3.8)
40–49	10 (9.5)
50–59	25 (23.8)
60–69	28 (26.7)
70+	23 (21.9)

*Tumour site*	
Breast	22 (21.0)
Gynaecological	23 (21.9)
Gastrointestinal	19 (18.1)
Prostate	4 (3.8)
Haematological malignancies	8 (7.6)
Lung	9 (8.6)
Other	6 (5.7)

*Treatment information*	
On treatment	17 (16.2)
Off treatment	58 (55.2)
Palliative (hospice)	14 (13.3)

Unavailable data are a result of a range of factors including data not being held at the participating site (such as the support group) or because participating sites were unable to provide the data.

**Table 2:** Research themes and ranked scores of research themes identified in consultation groups

**Rank**	**Key theme**	**Total rank score (possible range: 1–102)**	**Number of consultation groups in which topic received at least one vote *N*=17**
1	Impact on life, how to live with cancer and related support issues	68	13
2	Risk factors and causes	58	12
3	Early detection and prevention	48	9
4	Research into general information needs (on cancer, treatment, research and access to)	34	11
5	Use and effectiveness of complementary and alternative therapies	30	7
6	General education of public about cancer	24	5
7	Research into different cancer and patient types	23	7
7	Research on treatment (curative treatment, treatment types and improvements)	23	5
7	Experiences and management of side effects	23	7
8	Organisation and funding of health and social care services	21	6
9	Coordination, impact and funding of research	19	4
10	Research into recurrence	11	3
11	General communication issues involving all parties	10	3
12	Accessing patients' views about cancer, services and research	9	2
13	Health and safety in the hospital	1	1

**Table 3:** Excerpts from transcripts illustrating rationale for research ideas and priorities identified in consultation groups

**Research priority**	**Subthemes**	**Illustrative quotes from consultation group transcripts^a^**
Research priority 1: impact on life, how to live with cancer and related support issues	• Psychological consequences • Self-help groups and peer support • Follow-up and after care • Impact on social • Functioning • Work and other financial impacts • Pain management • Impact on family and others • Diet and other issues in managing cancer	‘It's not just about getting a stethoscope or dissecting things and looking at it, it's about things like aromatherapy and counselling for people with progressive or terminal illnesses. So it's not just about the actual tumour or the after effects of your operation, it's more, it's how you feel, whether or not you get depression, whether or not you're worried if it's going to go onto your family, all of that needs to be researched.’ (Liz, Colon Cancer Patient, Mixed Consultation Group) ‘I think money has to be a priority for research because, at the end of the day, we can't exist without finances and it is bad enough having cancer but there is probably only one thing worse than having cancer and that is having cancer with no money.’ (Kevin, Lung Cancer Patent, Mixed Consultation Group) ‘I would like more research done into [diet], just sensible suggestions, maybe unproven scientifically… the information I'm picking up, increasingly I think it will become one of the major ways of preventing as well as controlling cancer and maybe, I'd like a booklet that would encourage people to go away and eat healthily.’ (Ingrid, Ovarian Cancer Patient, Mixed Consultation Group)
Research Priority 2: Risk factors and causes	• Environmental • Genetic • Diet • Stress • Other	‘There must be research into electricity pylons. I know a particular pylon where I used to live and loads of people contracted cancer... What's in the ground, is there a stream with something running through I don't know?’ (Jim, Bowel Cancer Patient, Mixed Consultation Group) ‘The hereditary thing worries me and I think it should be researched… I have three grandchildren and I have been questioned, “Is this hereditary?” You know, I can't answer that.’ (Stephanie, Bowel Cancer Patient, Mixed Consultation Group) ‘My aunty had a bad car accident with her neck years ago, when there weren't any seatbelts and she developed throat cancer, so you often wonder whether trauma caused it.’ (Sophie, Breast Cancer Patient, Mixed Consultation Group)
Research Priority 3: Early detection and prevention	• Early diagnosis, detection and prevention. • GP awareness, knowledge and training, and related issues. • Means of prevention (e.g. diet)	‘Far better for a very long term outlook than fiddling around with cancer treatments. Sufferers as we are, we're interested in having research into effective treatments as well, but I think a fair bit of research money was directed into causes and therefore prevention of cancer by trying to eliminate the causes or educate people to avoid the causes.’ (Susan, Ovarian Cancer Patient, Mixed Consultation Group) ‘Well, I would also add that we could have more research into diet, because we read, if you eat 4lbs of beetroot every day, then it stops this, that and the other. A lot of it's rubbish, but is there any sort of research into diet that could be beneficial?’ (Elizabeth, Patient receiving palliative care services, Palliative Care Consultation Group)
Research Priorities 4 - 15	• General information needs • Use and effectiveness of CAM • General education of public • Research into different cancer and patient types • Research on treatment • Experiences and management of side effects • Organisation and funding of health and social care services • Coordination, impact and funding of research • Research into recurrence • General communication issues • Accessing patients' views about cancer, services and research • Health and safety	‘Ovarian cancer is supposed to be without any symptoms, but it isn't and I think if you asked a hundred people who have had ovarian cancer, “What exactly did you feel like, and what happened to you?”, and they'd say about the weight increase and you might get some idea of what the symptoms are. I don't believe it's without symptoms. I know it isn't and I think it's only education and publicity that will do this.’ (Tracy, Ovarian Cancer Patient, Mixed Consultation Group) ‘I hate to use the cliché, but something like looking out for new cures, sort of blue sky research. I think that's important.’ (Philip, Patient receiving palliative care services, Palliative Care Consultation Group) “There's such a plethora of research bodies, why isn't there a national integration? It's like anything else, they're all vying for one thing… why cannot we draw the line together and have some sort of national co-ordinating body that directs research where it should take place and get an equitable playing field?' (Colin, Pancreatic Cancer Patient, Mixed Consultation Group) “I think you've got to be careful to centralise, if you pool resources, then the trouble is you don't necessarily get the diversity of ideas that are being put forward. If you start to centralise things too much, then lateral thinking can go out the window, because one research centre will pursue one line and another one will pursue another. Now one might be no good, but if they're all doing the same thing and it was the wrong idea, you know.' (Steven, Bowel Cancer Patient, Mixed Consultation Group) “Maybe research [should go] into why you get a reoccurrence… why does it reoccur, maybe twenty years later or fifteen years later?’ (Kirsty, Breast Cancer Patient, Mixed Consultation Group)

a Pseudonyms are used in place of participant names throughout this paper.
